# Getting Back to Nature: Feralization in Animals and Plants

**DOI:** 10.1016/j.tree.2019.07.018

**Published:** 2019-12

**Authors:** Eben Gering, Darren Incorvaia, Rie Henriksen, Jeffrey Conner, Thomas Getty, Dominic Wright

**Affiliations:** 1 Department of Integrative Biology and Ecology, Evolutionary Biology, and Behavior Program, Michigan State University, East Lansing, MI, USA; 2 Department of Biological Sciences, Halmos College of Natural Sciences and Oceanography, Nova Southeastern University, Davie, FL, USA; 3 IIFM Biology and AVIAN Behavioural Genomics and Physiology Group, Linköping University, Linköping, Sweden; 4 Kellogg Biological Station and Dept. of Plant Biology, Michigan State University, Hickory Corners, MI, USA

**Keywords:** feralization, domestication, adaptation, invasion, admixture, evolution

## Abstract

Formerly domesticated organisms and artificially selected genes often escape controlled cultivation, but their subsequent evolution is not well studied. In this review, we examine plant and animal feralization through an evolutionary lens, including how natural selection, artificial selection, and gene flow shape feral genomes, traits, and fitness. Available evidence shows that feralization is not a mere reversal of domestication. Instead, it is shaped by the varied and complex histories of feral populations, and by novel selection pressures. To stimulate further insight we outline several future directions. These include testing how ‘domestication genes’ act in wild settings, studying the brains and behaviors of feral animals, and comparative analyses of feral populations and taxa. This work offers feasible and exciting research opportunities with both theoretical and practical applications.

## Highlights

Feral animals and plants have become ubiquitous worldwide, but their evolution has not been well studied.
The process of feralization offers unique and important opportunities to study adaptive evolution, often in model systems inhabiting diverse, novel, and/or changing environments.
Recent work shows that feral taxa undergo rapid evolutionary changes at loci controlling an array of fitness-related traits, including morphology, behavior, and development.
Gene flow between domesticated and wild populations has important, diverse, and context-dependent effects on fitness in recipient populations.
Legacies of domestication are seen in many feral plants and animals. These features can have important and unexpected roles in subsequent adaptation to changing (e.g., feral) environments.

## Domestication Is Not a Dead End

Domesticated animals and plants comprise a rapidly growing proportion of life on our planet [1]. The vast ranges and abundance of these organisms show that **domestication** (see Glossary) can have remarkable evolutionary payoffs. At the same time, it can induce both plastic and genetic modifications that limit the capacity of an organism to thrive in nature (e.g., 2, 3, 4). Despite this maladaptation, **feralization** of animals and plants has proven, sometimes to humans’ great frustration, that domestication is not always a one-way process. The flow of domesticated organisms and their genes into noncaptive settings has important conservation implications; it also presents unique opportunities to characterize general and novel evolutionary processes of Anthropocene environments [5]. With these applications in mind, our review summarizes current knowledge regarding the process of feralization and provides a roadmap for further investigation into this tractable, exciting, and understudied research area.

Feralization merits special consideration because its subjects are uniquely distinguished from other animals and plants. Biologists have long appreciated how domestication shapes wild organisms via both deliberate **artificial selection** by humans and unintended effects of anthropogenic propagation [6]. In recent decades, these effects have been elucidated by intensive studies bridging disparate fields (e.g., anthropology, plant and animal science, and organismal, behavioral, and developmental biology) 7, 8, 9. By contrast, there has been relatively little research into the process of feralization. Here, progress is also hindered by long-held speculations and misconceptions. These include: (i) the idea that formerly domesticated populations are incapable of rapid adaptation, due to their genetic homogeneity or recent establishment [10]; (ii) the idea that captive propagation invariably reduces **fitness** outside of domesticated settings due to evolutionary tradeoffs and relaxed natural selection (e.g., 2, 11); and (iii) a belief that feralization predictably results in **atavism** (e.g., [12]). These ideas have received only mixed support from a small but growing body of relevant research. Here, we draw on case studies to: (i) show that routes to feralization are diverse and can facilitate rapid evolution; (ii) synthesize current knowledge concerning feral genotypes and phenotypes; and (iii) outline avenues for future studies.

## Pathways to Feralization

### Defining Domestication and Feralization

There are many extended discussions of problems surrounding the definition of domestication (e.g., 13, 14, 15). The broadest definitions encompass nonhuman species, such as leaf-cutter ants, that also cultivate mutualists (e.g., [16]). Yet, while these cultivars can feralize [17], such non-anthropogenic processes lie beyond the scope of this review. Others 13, 18 describe domestication as movement along continua of human–animal interactions or, alternatively, as solely the onset of human-facilitated propagation (e.g., [11]). In this review, we expand an operational definition developed for animals [19] to include agricultural and ornamental plants. Except where noted otherwise, we also adopt the inclusion by this definition of both the establishment and subsequent improvement stages of anthropogenic propagation.

Our review also examines how the allele frequencies, traits, and fitness of wild populations can be altered by the **introgression** of **feral alleles** from artificially selected sources; thus, it encompasses many wild gene pools that are chiefly derived from undomesticated ancestors 20, 21. Here, we show that even limited introgression from artificially selected sources can have important evolutionary consequences. For clarity, however (except where noted), we use ‘feral’ to describe free-living organisms or populations that are primarily descended from domesticated ancestors.

**Figure 1:**
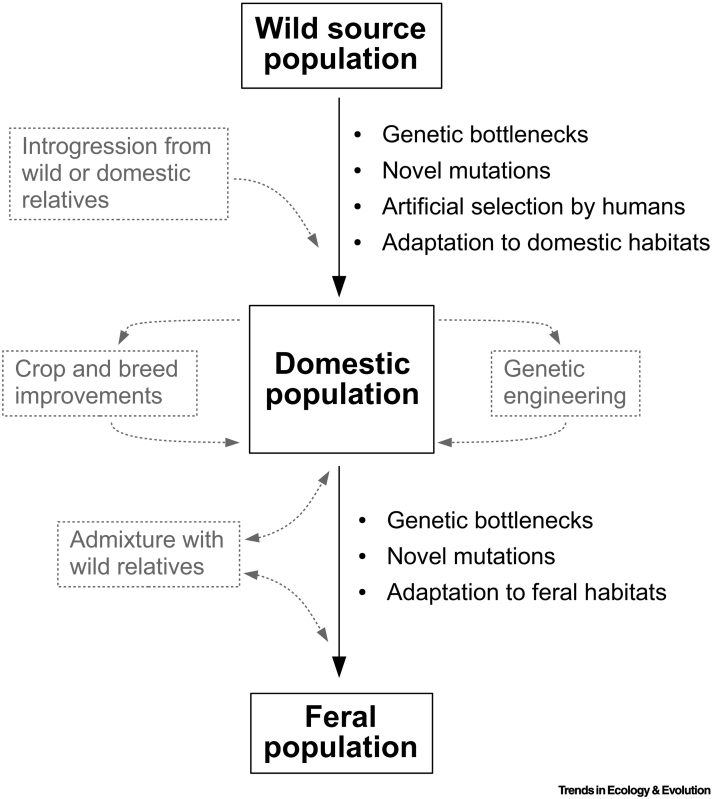
Evolutionary Forces That Shape Feral Gene Pools and Traits. The core process of feralization (depicted here with solid black arrows and boxes) is often modified by various forms of gene flow and/or anthropogenic selection (depicted here as dashed gray lines and boxes). For a Figure360 author presentation of Figure 1, see the figure legend at https://doi.org/10.1016/j.tree.2019.07.018 Figure360: An Author Presentation of Figure 1

Our discussion of feralization requires a few caveats. First, some **feral populations** still receive limited, intentional support from humans. For example, feral cats and horses are sometimes provisioned with food, yet remain highly self-reliant compared with their domestic counterparts and do not fulfill an artificially selected utility. Additionally, some taxa have oscillated between feral and domestic states, blurring lines between the two processes (e.g., longhorn cattle that were redomesticated from feral ancestors) [22]. Finally, we acknowledge that feralization need not involve a return to truly ‘wild’ habitats. Instead, it often unfolds within cultivated or disturbed settings (e.g., agricultural fields and cities). Still, its subjects are distinguished from domesticated ancestors by the withdrawal of intentional efforts to support their reproduction. This alters selection regimes in ways that can, both in principle and practice, produce rapid evolutionary changes (Figure 1).

### Sources of Feral Populations

To understand how populations evolve, it is usually helpful to examine their sources and genetic structures. Given that feral populations compound demographic and selective effects of domestication with a subsequent ‘re-invasion’, they present unique challenges for DNA-based ancestry reconstructions, as well as for sequence-based tests of adaptation 4, 23. Despite these obstacles, many investigators have succeeded in elucidating pathways to ferality. Gressel [24] delineated two alternative categories, which we illustrate with diverse examples in Table 1. ‘Endoferal’ populations stem from a single domesticated lineage (e.g., a breed or crop), whereas ‘exoferal’ populations are derived via **admixture**, either among domesticated lineages (e.g., crop varieties) or between domestic taxa and their wild relatives. Current data suggest that both endo- and exoferality are common. Among 23 plants that have feralized into weedy or invasive forms, approximately equal numbers were found to involve endo- versus exoferal origins [25]. Both mechanisms have also produced feral animal populations (Table 2), although their relative roles have not been systematically reviewed.

**Table 1:** Animal and Plant Domestications That Have Resulted in Feralization, and Their Primary (Artificially Selected) Utilities

Order	Domesticated taxon		Antiquity (years before present)	Food	Companionship	Aid	Security	Ornament	Sport-racing	Warfare	Sport-fighting	Transport or draft	Textiles	Pest control	Pollination
Mammals															
Carnivora	Dog, dingo	*Canis lupus*	15 000^a^												
	House cat	*Felis catus*	9500^a^												
	American mink	*Neovison vison*	80^b^												
Lagomorpha	Rabbit	*Oryctolagus cuniculus*	1300–17 000^c^												
Perissodactyla	Pig	*Sus scrofa*	10 300^a^												
	Horse	*Equus ferus*	5500^a^												
	Ass	*Equus africanus*	5500^a^												
Artiodactyla	Goat	*Capra aegagrus hircus*	10 000^a^												
	Sheep	*Ovis aries*	10 000^a^												
	Cow	*Bos taurus*	10 300^a^												
	Dromedary camel	*Camelus dromedarius*	3000^a^												
Birds															
Galliformes	Chicken	*Gallus gallus*	4000^a^												
	Turkey	*Meleagris gallopavo*	2000^d^												
Columbiformes	Street pigeon	*Columbus livia*	>5000^b^												
Anseriformes	Mallard	*Anas platyrhynchos*	1000^a^												
	Muscovy duck	*Cairina moschata*	Pre-Columbian												
Insects															
Hymenoptera	Honeybee	*Apis mellifera*	9000^e^												
Lepidoptera	Silkworm	*Bombyx mori*	7500^e^												
Fish															
Salmoniformes, Cyprinodontiformes, Cypriformes, Cichliformes, Anabantiformes	Aquacultural and pet species	e.g., salmon, cichlids, guppies, betas	Variable												
Plants															
Asterales	Jerusalem artichoke	*Helianthus tuberosus*													
Poales	Bread wheat	*Triticum aestivum*	10 000^f^												
	Finger millet	*Eleusine coracana*	5000^f^												
	Grain sorghum	*Sorghum bicolor*	5000^f^												
	Rice	*Oryza sativa*	7000^f^												
	Rye	*Secale cereale*	5000^f^												
Brassicales	Radish	*Raphanus raphanistrum*	8000^g^												
Caryohyllales	Sugarbeet	*Beta vulgaris*	300^f^												

a From [100].

b From [101].

c From [102].

d From [103].

e From [104].

f From [105].

g From [106].

**Table 2:** Sources of Feral Animals and Plants

Domestic population crossed with	Definition^a^	Examples
Self	Endoferal	Crop rice (*Oryza sativa*) appears to be particularly prone to feralization, because there is evidence for multiple de-domestication events with varying origins in Asia and North America. Weed rice populations of endoferal origin are present on both continents [98]. Endoferality is common in animals, including serial introductions of rabbits to Australia that have generated genetically distinct endoferal subpopulations [47]
Divergent population (e.g., breed or crop)	Exo–endoferal (intercrop)	In Bhutan, weedy rice is a hybrid of two crop varieties (*O.s. japonica* × *O.s. Indica*) [98]. Feral cattle in the New World that were subsequently re-domesticated stemmed from admixture between independently domesticated taurine and indicine aurochs (*Bos primigenius*), and this admixture may have facilitated adaptation to novel environments outside the native range [22]
Wild conspecific	Exoferal (crop–wild)	SNP diversity of weedy rice is higher in southwest Asia than in the range of wild rice, due to introgression from wild rice and also perhaps from local crop rice landraces [98]. Exoferal (domestic–wild) animals include chickens that hybridize with red junglefowl (*Gallus gallus*) within the native and introduced ranges of the species 30, 107
Other domesticated species	Exoferal (domestic hybrid)	Feral Jerusalem artichoke (*Helianthus tuberosus*) and domesticated sunflower (*Helianthus annuus*) may hybridize in Europe [25]
Other wild species	Exoferal (crop–wild hybrid)	California wild radish is an interspecific hybrid between the crop radish (*Raphanus sativus*) and the agricultural weed ecotype of native wild radish (*Raphanus raphanistrum*; [64]). Available evidence suggests that the agricultural weed radish is derived from the native wild radish [106]. Animal examples are rare, but include coyote–dog (*Canis latrans* × *C. lupus*) hybrids [58]
Genetically modified organism	Exoferal (transgene hybrid)	Transgenes have been found in several wild plant populations 37, 38, 39, 40. Animal cases are not yet known, partly due to legal, logistical, and technological barriers to the cultivation of transgenic animals

a After 24, 25.

### Mechanisms of Feralization

Endoferality can occur when individuals from a domestic population escape into local environments in which they can survive and reproduce. This is what most people envision when contemplating feralization. Endoferality can also result from intentional releases of organisms to establish feral descendants. We call this process ‘de-domestication’ (*sensu* [15]), although the term is used in the plant literature synonymously with atavism (e.g., [26]). Motives for releases of domestic taxa range from ecosystem engineering [27] to providing recreational, nutritional, and/or economic benefits (e.g., hunting and fishing) [28].

Exoferality, by definition, involves admixture. Sometimes, this gene flow precedes translocation into new environments, as shown by a subset of North American weedy rice that originated from admixture outside of their introduced range [29]. Admixture can also occur at multiple timepoints during and after establishment. For example, archeological, morphological, and genetic evidence suggest that, centuries after Polynesians dispersed red junglefowl (*Gallus gallus*) into Pacific Oceania, the descendants of these birds hybridized with chickens introduced by Europeans (e.g., 30, 31, 32, 33). These and other exoferal populations (e.g., Table 2) provide tractable systems for studying how gene flow impacts the establishment, fitness, and local adaptation of non-native organisms, a central goal of invasion biology (e.g., 4, 23, 34, 35, 36). In addition, a subset of exoferal gene pools harbor feralized **transgenes**, an increasingly common phenomenon that raises unique ethical issues and research questions [37]. Transgenes have introgressed into nonagronomic plant populations (e.g., wild cotton and bentgrass 38, 39), into cultivated crops (e.g., canola, soybean, and maize [40]), and into feral plants (e.g., weedy rice and beets 41, 42). Thus, gene flow among domestic, feral, and wild plants comprises an important potential mechanism for transgene establishment and spread.

In the near future, broadening of sampling and analytical tools will likely increase the number of feral populations with known exoferal origins [23]. Ancient DNA can also be used to clarify population ancestries (e.g., 43, 44). Recently, for instance, this approach revealed that modern Przewalski’s horses are in fact feral descendants of horses domesticated by the Botai culture, rather than truly wild [45]. Furthermore, recent introgression from domestic horses has introduced deleterious gene variants to this exoferal gene pool.

The diversity of pathways to feralization (Table 2) raises an interesting issue regarding the modeling of the process. Although endoferal populations provide the clearest insights into how feral selection regimes affect formerly domestic gene pools and traits (i.e., evolution *in absentia* of admixture), they may also represent a minority of feralization episodes in nature. A parallel conundrum has catalyzed recent revisions of domestication models, since the process involves admixture more often than previously thought, and it can also be difficult to detect [8].

Viewing feralization ‘in light of admixture’ helps to clarify how future gene flow can impact outcomes and consequences of the process. For example, many feral taxa (e.g., weedy rice, dogs, and chickens) appear to exhibit both exo- and endoferal origins across their current ranges. These interpopulation differences result in both genetic and phenotypic variation (e.g., 25, 30, 46, 47), which would likely be affected by further introgression (e.g., admixture between genetically divergent feral populations; e.g., [29]). Admixture from domestic sources can also convert wild populations into exoferal ones [20] and accelerate their responses to new selection pressures [48]. Remarkably, genes from 23 of humanity’s 25 most important domesticated plants have been found in wild populations. The geographical distribution and phenotypic consequences of this crop–wild admixture vary widely by case [49]. The same phenomenon is seen in animals, with examples including wolf × dog, chicken × red junglefowl, and farmed × wild salmonid hybrids. We briefly explore the fitness effects of these exchanges in the following section.

## Adaptation in Feral Organisms

### Fitness Consequences of Admixture

Several methods are available for assessing how admixture affects fitness in feral populations, including: (i) direct measurements of growth, survival, reproduction, and health in hybrids; (ii) functional analyses of outlier loci detected in genome scans (e.g., 50, 51); and (iii) experimental tests of the effects of these loci in laboratory systems (e.g., [50]). In recipient wild populations of fish, these approaches often find outbreeding depression (e.g., 52, 53). Reductions in hybrid fitness are also seen in weedy plants (e.g., [54]). These patterns can arise through the disruption of coadapted genes, allelic incompatibilities between source populations, and/or when gene variants from one source (e.g., domestic settings) are locally maladaptive in ferals 4, 55, 56. Altogether, this may explain why recipient wild populations often contain a small fraction of genes from domestic sources. Animal examples in which domestic introgression is minor (∼5–10%) include wolves (e.g., 57, 58, 59), wild boar 60, 61, coyotes 58, 62, and partridges [63].

However, in some situations, exoferal hybrids can have higher fitness than source populations. In greenhouse common gardens, functional traits of California wild radish were either phenotypically intermediate between the source populations of these hybrids or ‘domestic-like’ [64]. In exception to this pattern, California wild radish fruits were heavier than either parental taxon [64], were better protected against house finch damage [65], and had higher fitness in three common gardens within the invasive range of the hybrid [66]. This apparent hybrid vigor may help explain the capacity of the exoferal hybrid to thrive in noncultivated habitats and displace both domestic and wild progenitors.

Alleles involved in domestication and improvement can also facilitate adaptation in animals. For example, admixture between independently domesticated cattle likely facilitated the adaptation of the longhorn to feral conditions within the New World [22]. In general, we suspect that alleles that were artificially selected to enhance production (e.g., accelerating growth or fecundity) may often prove beneficial in nature, particularly during the establishment and expansion of feral populations (e.g., 50, 67). Still, more work is needed that examines the genetic basis of fitness-related **phenotypes** in feral settings. These studies should also compare genotype–phenotype relationships across populations and/or conditions, because hybrid fitness can vary sharply between environments (e.g., in carrots, radish, and salmon 25, 68, 69), and because plasticity can be important in colonizing novel ones 34, 70. Thus, accounting for gene × environment interactions will be essential for forecasting future feralization trajectories in the variable and changing environments of the Anthropocene 5, 71.

### Effects of Domestication and Improvement Alleles

Domestication has produced consistent, correlated changes in a variety of species, such that **domestication syndromes** are commonly observed in both animals and plants [72]. The genetic mechanisms that produce these shared phenotypes within evolutionarily distant taxa is an area of intensive current research. In animals, one hypothesis proposes that syndromes arise through correlated effects of tameness selection on the development of neural crest-derived cells 73, 74. This idea is supported by emerging findings of parallel evolution in pathways that control neural crest cell fates in distantly related taxa (e.g., 59, 75). Plant domestication syndromes involve an array of traits, including attenuated seed dormancy and dispersal, vertical growth forms, increased seed size, accelerated growth, and palatability [11]. As in mammals, many of these traits involve complex gene networks and biochemical pathways that are evolutionarily conserved in distantly related taxa. At present, the extent to which domestication modifies homologous genomic loci to produce animal and plant domestication syndromes is not clear (e.g., [76]). Fortunately, emerging discoveries within this area (e.g., 73, 77) will soon enable us to determine whether (and how) domestication syndromes evolve under feralization.

In addition to exhibiting similarities in the form of syndromes, domesticated taxa are also differentiated from one another by their unique ancestries, cultivation or husbandry methods, and artificially selected utilities. Central goals in domestication research are to determine: (i) which genetic changes were directly selected by humans; and (ii) which variants and traits were crucial for the onset of domestication [11]. However, to understand feralization, it is important to examine the frequencies and functions of both **domestication** and **improvement alleles**. Together, these features distinguish contemporary domestics from their wild relatives, and we suspect that they can both contribute to the local adaptation or maladaptation of feral populations.

Table 3 provides diverse examples of loci with major effects on domestic phenotypes. In domesticated settings, functional impacts of these genes are sometimes known. By contrast, their allele frequencies and phenotypic effects are largely unstudied in feral populations. This offers compelling directions for future research, including determining the significance of: (i) mutations and structural variants arising *de novo* within domestic populations (versus ancestral variants recruited by soft sweeps or drift); (ii) gene variants affecting protein structures and gene expression; and (iii) fitness consequences of domestication versus improvement alleles. Expanding this work to include polygenic traits will be similarly important for understanding feralization, because many domestication-related phenotypes are only partly attributable to loci of major effect 78, 79. However, these are more technically challenging to characterize, and further work is first needed to elucidate their modification by domestication.

**Table 3:** Examples of Loci Involved in the Domestication or Subsequent Improvement of Plant and Animal Morphology and Physiology, and Their Significance to Feralization

Trait	Gene(s)	Domestic phenotype	Domesticated variant present in ferals	Fitness effects in the wild	Refs
Animals					
Morphology	*TYRP1*	Melanic coat color in sheep	+	Artificially selected ‘light color’ phenotype was positively selected in feral Soay sheep	[108]
	*CBD103*	Melanic coat color in wolves	+	A continent-wide selective sweep in wolf × dog hybrids may result from the domesticated variant enhancing survival	[109]
	*MC1R*	Coat color in pigs	+	Domestic phenotypes involving this locus are common in Pacific feral pigs, perhaps indicating relaxed or positive selection	[61]
	*RXFP2*	Horn type in sheep (normal or scurred)	+	In feral Soay sheep, male heterozygotes have high fitness due to a balance of sexual costs and longevity gains of an artificially selected allele producing smaller horns. *RXFP2* genotypes were not found to affect female survival or fitness	[110]
Growth and physiology	?	Increased fecundity in pigs	+	Domesticated gene variants may increase fecundity in admixed wild populations near farms. This example highlights the many cases where causal genes are not yet known	[60]
	*IGF1*, *GHR*, IGFII, *THR*	Increased growth in Salmon	?	Effects of alleles from wild-type, domestic, and/or transgenic origin can vary across environments. Domesticated alleles are often deleterious	[111]
Plants					
Growth and physiology	*SH4*, *qSH1*	Delayed seed shattering in rice	+	Domesticated phenotype is absent in weedy derivatives of domestic rice, although they do carry the domesticated allele at *sh4*. Compensatory mutations may have been positively selected to facilitate weediness	78, 112
	*CBF*	Stress tolerance in barley	?	Unknown, but may affect abiotic stress tolerance. *HvCBF4* is important for salt tolerance in wild Tibetan barley, the source of domesticated barley	[113]
	*FRI*	Flowering time in rapeseed	?	Unknown, although multiple orthologs are important for flowering time in rapeseed (*Brassica napus*)	[114]
	?	Life history and morphology	+	A mixture of crop and wild traits were positively selected in outplanted hybrid sunflowers	[115]

Another novel and potentially transformative goal for future studies is to characterize structural and functional properties of feral microbiomes, which affect an array of fitness-related traits and can evolve rapidly during feralization [80]. For example, even after many generations outside of captivity, feral chickens retain legacies of captive husbandry within their digestive microbiota (e.g., a somewhat attenuated resistance to agroindustrial antibiotics). Nonetheless, these feral microbiomes are also both divergent from, and more variable than, those of farmed poultry reared on a variety of diets [80]. The causes and consequences of microbiome divergence have broad basic and applied significance, and merit further (e.g., comparative) analyses.

### Direct Observations of Selection in Feral Populations

One of the most powerful tools for identifying adaptive changes during feralization is to analyze long-term pedigrees; an island population of Soay sheep studied since the 1960s offers one example [81]. In this case, pedigrees were used to infer the selection pressures on several phenotypes with domestic origins. Here, a genetic polymorphism affecting coat color is known, with the heritable black phenotype having a large body size and higher fitness [82]. However, due to the linkage between a major gene for black coloration and a quantitative trait locus (QTL) with antagonistic effects on size and fitness, black coloration is declining in this population.

## Plasticity and Reversion of Feral Traits

### Feral Brains and Behaviors

**Phenotypic plasticity** can be crucial in the colonization of novel environments [70]. Animal brains are of central importance for behavioral plasticity, and many domestic animals have diminished brain volumes [83]. This pattern is attributed to the relative simplicity of domestic environments [84], to artificial selection for docility and tameness, and to correlational selection on other traits [85]. Thus, feralization offers unique opportunities to study how brains and behavioral traits evolve when domestic animals transition into highly heterogenous and unpredictable environments.

Table 4 lists several known features of the brains and behaviors of domestic and feral animals. Somewhat surprisingly, many studies have found no effect of feralization on brain volumes 86, 87. Here, evolution may be hindered by a lack of essential genetic variation or insufficient time. The latter hypothesis is consistent with findings from dingoes, which are likely among the oldest feral populations (since ∼3000–8600 y before present). Dingo brains are larger and more encephalized than those of domestic dogs of similar body size, although variation among dog breeds complicates these comparisons [88]. Feralization may also drive subtler changes in brain structure and function. For instance, pigs were released on the Galapagos Islands ∼100 years ago to serve as meat reserves. Over the decades that followed, proportional sizes of differently-specialized brain regions diverged from those of domestic pigs [89]. Effects of domestication and feralization on brain function are also evident in molecular data, including: (i) comparative studies of domestic mammals revealing divergence in brain-specific miRNAs [90]; and (ii) evidence of selective sweeps at loci controlling neuronal development in feral chickens [50].

**Table 4:** Effects of Domestication and Feralization on Behavior-Related Phenotypes^a^

Behavioral trait	**Δ** Domestic (versus wild) phenotype		**Δ** Feral (versus domestic) phenotype	
Brain volume	↓	Diverse mammals, birds, fish [86]	=	Diverse mammals [86], with exception of dingo [88]
Proportional size of brain regions	↕	Altered allometry of motor, limbic, and sensory regions in diverse taxa. Most pronounced regressions affect limbic regions [86]	↕	In exception to many examples of stasis [86], dingoes and pigs show partial ‘wild-type’ reversions 88, 89
Gene expression in brain	↕	Dogs [116], cows, horses, pigs, rabbits [90]	?	
Aggression toward conspecifics	↕	Reduced agonism in many taxa, including fish and dogs. Increased agonism in some fighting breeds (e.g., bulls and cockerels [12])	↑	Roosters [92]
Predator avoidance	↓	Chickens, pheasants, rodents, fish 19, 86, 93, 117	↑	Chickens [92], guppies [93]
Habitat selectivity	↓	Deer mice [12]	?	
Neophobia	↓	Mice, rats [19]	↑	Chickens [92]
Stress response	↓	Guinea pigs, foxes, mice [116]	?	
Reproductive seasonality	↓	Foxes [116], chickens [118], dogs [19]	?	
Diet selectivity	↓	Cats [86]	↓	Salmon parr [12]
Vocalization	↕	Higher rates in dogs, birds, guinea pigs [12], reduced diversity in birds [19]. Rates are also variable among breeds [117]	?	

a ↑trait magnitude is higher; ↓trait magnitude is lower; ↕trait change varies by case (e.g. among previously-studied taxa, contexts, or populations).

At the level of behavior, domestication has often reduced fearfulness, agonism, and overall behavioral responsivity 19, 91; these effects can also be modified in ferals. For example, feral roosters, quails, and guppies were found to be more fearful, agonistic, and alert to potential predators compared with domestics 92, 93 (C.R. Nichols, PhD thesis, University of British Columbia, 1991). There are many other known differences between the social behavior and communication of feral animals and domestics (e.g., 30, 94) (Table 4). Both plasticity (e.g., learning) and genetic evolution can impact these traits [19] and their relative roles have not been systematically examined. Furthermore, fitness consequences of behavioral variation in feral populations remains poorly studied.

### Other Feral Traits

While we have emphasized behavioral traits in the preceding section, animal and plant morphology and physiology have, likewise, been profoundly altered by domestication. By way of example, domestication has altered plant chemical defenses mediating herbivory in cultivated and wild settings [3]. These changes, and possibly subsequent ones, likely impact fitness in feral plants, although this has not yet been studied. Alongside many other examples of morphological and/or physiological trait change (e.g., Table 3), this shows how feralization research could both deepen, and expand upon, ecologically enlightened views of the fitness consequences of domestication [3].

### Reconsidering Reversions

Many early naturalists reported that feral organisms invariably revert to the ‘wild-type’ traits of their ancestors. While Darwin took interest in the atavism of feral domestics, he also questioned its ubiquity [6]. Today, genomic studies are proving, intriguingly, that even when feralization restores ancestral phenotypes, this reversion can involve novel genetic mechanisms. For example, grain crops have been selected by humans to retain seeds until their harvesting. Given that seed dispersal is a crucial adaptation for most wild plants, reversion to dispersive phenotypes should be common in feralized grain crops. Seed dispersal in rice is called shattering, and this trait has been well studied in weedy rice. A key gene in the decreased shattering of domesticated rice is *sh4* [95], but reversions to a shattering phenotype in US weedy rice are not caused by changes at this same locus [96]. Rather, they are controlled by different genomic regions in each of the two weedy rice groups, suggesting independent restorations of a ‘wild-type’ trait [97]. By contrast, in Southeast Asia, shattering in weedy rice is caused at least in part by adaptive introgression of wild alleles at *sh4* [98]. Finally, in feral chickens and sheep 50, 51, genome scans found only limited overlap between outlier loci (i.e., candidate ‘feralization loci’), and genome regions that are known to have evolved under domestication. Altogether, these examples show that, at the genetic level, domestication-related changes are not predictably reversed by feralization. In systems where phenotypic reversion has occurred despite this (e.g., in weedy rice), we can now begin to disentangle how stochastic factors, the reversibility or irreversibility of evolution, and/or differences between ancestral and feral environments (e.g., emergent competition with domesticated counterparts [99]) steer the process of feralization.

## Concluding Remarks and Future Directions

There is ample evidence that the evolution of feral populations is shaped by their unusual environments and histories. However, a robust understanding of feralization necessitates more studies that elucidate causal roles of selection pressures and genetic variation in the evolution of feral traits and fitness. A search for convergent ‘feralization syndromes’ could help illuminate proximate and/or ultimate mechanisms that drive feralization. At the same time, the process of feralization itself will continue to evolve. For example, genome editing is poised to alter domestication processes, and may generate novel feral populations as a byproduct [4].

In addition to providing Outstanding Questions, we close with some limitations of prior studies. First, many researchers have compared feral taxa to domestic relatives that are not their original source population(s). Therefore, differences in phenotypes and genotypes cannot be conclusively attributed to feralization. Furthermore, few studies have explicitly accounted for effects of differing methods and objectives of artificial selection (e.g., Table 1) on descendent feral populations. Lastly, the literature contains few comparative studies across feral populations or species. Nonetheless, the fact that feralization has often occurred to the same domesticated species in separate parts of the world offers opportunities to identify the constraints and pressures, be they environmental or genetic, that shape the course of feralization. After decades of intensive study, domestication research continues to provide stunning and practical evolutionary insights. Clearly, the open frontiers of feralization research hold equally exciting prospects for investigators bold enough to venture beyond the farm (see Outstanding Questions).

Outstanding Questions How predictable is feralization? We will need more comparisons of gene pools and traits (both among populations and between species) to answer this question, which has both applied and conceptual significance.

What genetic mechanisms drive feralization processes? Selection scans find evidence of rapid evolution in feral genomes, but have limited ability to detect many kinds of change that could impact feralization (e.g., structural rearrangement, epistasis, soft sweeps, balanced polymorphism, and heritable epigenetic change).

How do social and natural selection act in feral settings? Findings of ‘feralization genes’ suggest compelling hypotheses about fitness effects in nature, but these effects remain largely untested in natural settings (especially in nonplant and -fish models).

How do gene × environment relationships influence feralization? Both theory and empirical studies are needed to better understand how heterogenous and/or changing environments affect formerly domesticated populations. One important variable is the microbiome, which can evolve more quickly than (feral) host genomes and impacts a multitude of functional traits.

How does admixture affect feral population fitness and persistence? Recent work shows that admixture (e.g., between domesticated lineages and wild relatives) can facilitate rapid evolution. Over longer timeframes, we also see significant variability in the dynamics and persistence of feral populations. We do not yet know if and/or how admixture contributes to this variability in feralization outcomes.

Can feralization syndromes be identified? If feral animals exhibit parallel distinctions from domestic and/or nondomesticated counterparts, we can test whether these involve homologous mechanisms (e.g., genes or pathways). This is among the most feasible, and potentially transformative questions on the near horizon of feralization studies.

Alt-text: Outstanding Questions

## Glossary

**Admixture**: genetic exchanges between divergent gene pools.
**Artificial selection**: human-directed propagation of organisms with heritable and desirable traits. Darwin called this ‘methodical’ selection.
**Atavism**: restoration of ancestral (e.g., ‘wild-type’) phenotypes.
**Domestication**: process by which human-propagated organisms adapt to humans and the environments they provide.
**Domestication alleles**: allelic variants responsible for the phenotypic divergence between domesticated taxa and their wild ancestors. Domestication alleles can originate from: (i) ‘soft selective sweeps’ of standing variation in wild source population; (ii) genetic introgression from other sources; or (iii) *de novo* point or structural mutations in germlines undergoing domestication.
**Domestication syndrome**: suites of correlated traits that distinguish domesticated animals and plants from wild relatives.
**Feralization**: process by which formerly domesticated organisms (or artificially selected gene variants) become established *in absentia* of purposeful anthropogenic propagation.
**Feral alleles**: gene variants that descend from a domesticated population.
**Feral population**: population that descends chiefly from artificially selected ancestors.
**Fitness**: relative or absolute rates of genetic propagation (e.g., into viable offspring) by individuals or populations.
**Improvement alleles**: allelic variants that are involved in anthropogenic modifications of domesticated plants and animals, including the specialization of breeds and crop varieties. Improvement alleles can arise through the same three mechanisms as domestication alleles, and also via genome editing.
**Introgression**: influx of genetic variation to a focal, recipient population from a divergent gene pool through hybridization and backcrossing of hybrids.
**Phenotype**: observable trait of an organism (e.g., aspect of morphology, behavior, or development).
**Phenotypic plasticity**: potential for an organism (i.e., genotype) to produce a range of phenotypes when induced to multiple environments (i.e., environmentally induced phenotypic variation).
**Transgene**: gene that has been artificially introduced to the genome of an engineered organism (e.g., livestock or crop species).

## References

[1] Bar-On Y.M. (2018). The biomass distribution on Earth. Proc. Natl. Acad. Sci. U. S. A..

[2] Araki H. (2009). Carry-over effect of captive breeding reduces reproductive fitness of wild-born descendants in the wild. Biol. Lett..

[3] Milla R. (2015). Plant domestication through an ecological lens. Trends Ecol. Evol..

[4] Gering E. (2019). Maladaptation in feral and domesticated animals. Evol. Appl..

[5] Sarrazin F., Lecomte J. (2016). Evolution in the Anthropocene. Science.

[6] Darwin C. (1868). The Variation of Animals and Plants under Domestication.

[7] Martin A., Orgogozo V. (2013). The loci of repeated evolution: a catalog of genetic hotspots of phenotypic variation. Evolution.

[8] Larson G. (2014). Current perspectives and the future of domestication studies. Proc. Natl. Acad. Sci. U. S. A..

[9] Brandenburg J.T. (2017). Independent introductions and admixtures have contributed to adaptation of European maize and its American counterparts. PLoS Genet..

[10] Allaby R.G. (2019). A re-evaluation of the domestication bottleneck from archaeogenomic evidence. Evol. Appl..

[11] Meyer R.S., Purugganan M.D. (2013). Evolution of crop species: genetics of domestication and diversification. *Nat. Rev. Genet*..

[12] Price E.O. (1984). Behavioral aspects of animal domestication. Q. Rev. Biol..

[13] Russell N. (2002). The wild side of animal domestication. Soc. Anim..

[14] Bökönyi S., Clutton-Brock J. (1989). Definitions of animal domestication. The Walking Larder. Patterns of Domestication, Pastoralism, and Predation.

[15] Gamborg C. (2010). De-domestication: ethics at the intersection of landscape restoration and animal welfare. Environ. Values.

[16] Zeder M.A. (2015). Core questions in domestication research. Proc. Natl. Acad. Sci. U. S. A..

[17] Mueller U.G. (2005). The evolution of agriculture in insects. Annu. Rev. Ecol. Evol. Syst..

[18] Ballard J.W.O., Wilson L.A.B. (2019). The Australian dingo: untamed or feral?. Front. Zool..

[19] Price E.O. (2002). Animal Domestication and Behavior.

[20] Randi E. (2008). Detecting hybridization between wild species and their domesticated relatives. Mol. Ecol..

[21] Canestrelli D. (2016). The tangled evolutionary legacies of range expansion and hybridization. Trends Ecol. Evol..

[22] McTavish E.J. (2013). New World cattle show ancestry from multiple independent domestication events. Proc. Natl. Acad. Sci. U. S. A..

[23] McFarlane S.E., Pemberton J.M. (2019). Detecting the true extent of introgression during anthropogenic hybridization. Trends Ecol. Evol..

[24] Gressel J. (2005). Crop Ferality and Volunteerism.

[25] Ellstrand N.C. (2010). Crops gone wild: evolution of weeds and invasives from domesticated ancestors. Evol. Appl..

[26] Wang H. (2017). Asian wild rice is a hybrid swarm with extensive gene flow and feralization from domesticated rice. Genome Res..

[27] Rubenstein D.R. (2006). Pleistocene park: does re-wilding North America represent sound conservation for the 21st century?. Biol. Conserv..

[28] McCann B.E. (2018). Molecular population structure for feral swine in the United States. J. Wildl. Manag..

[29] Londo J.P., Schaal B.A. (2007). Origins and population genetics of weedy red rice in the USA. Mol. Ecol..

[30] Gering E. (2015). Mixed ancestry and admixture in Kauai’s feral chickens: invasion of domestic genes into ancient Red Junglefowl reservoirs. Mol. Ecol..

[31] Thomson V.A. (2014). Using ancient DNA to study the origins and dispersal of ancestral Polynesian chickens across the Pacific. Proc. Natl. Acad. Sci. U. S. A..

[32] Cornwallis C. (2002). The status and degree of hybridisation of Red junglefowl on three islands – a comment. Tragopan.

[33] Peterson A.T., Brisbin I.L. (2005). Phenotypic status of Red Junglefowl *Gallus gallus* populations introduced on Pacific islands. Bull. Br. Orn. Club.

[34] Whitney K.D., Gering E. (2015). Five decades of invasion genetics. New Phytol..

[35] Welles S.R., Dlugosch K.M., Rajora O. (2018). Population genomics of colonization and invasion. Population Genomics.

[36] Bock D.G. (2015). What we still don’t know about invasion genetics. Mol. Ecol..

[37] Ellstrand N. (2018). ‘Born to run’? Not necessarily: species and trait bias in persistent free-living transgenic plants. Front. Bioeng. Biotechnol..

[38] Wegier A. (2011). Recent long-distance transgene flow into wild populations conforms to historical patterns of gene flow in cotton (*Gossypium hirsutum*) at its centre of origin. Mol. Ecol..

[39] Reichman J.R. (2006). Establishment of transgenic herbicide-resistant creeping bentgrass (*Agrostis stolonifera* L.) in nonagronomic habitats. Mol. Ecol..

[40] Mallory-Smith C., Zapiola M. (2008). Gene flow from glyphosate-resistant crops. Pest Manag. Sci..

[41] Chen L.J. (2004). Gene flow from cultivated rice (*Oryza sativa*) to its weedy and wild relatives. Ann. Bot..

[42] Darmency H. (2007). Transgene escape in sugar beet production fields: data from six years farm scale monitoring. Environ. Biosaf. Res..

[43] Almathen F. (2016). Ancient and modern DNA reveal dynamics of domestication and cross continental dispersal of the dromedary. Proc. Natl. Acad. Sci. U. S. A..

[44] Ottoni C. (2017). The palaeogenetics of cat dispersal in the ancient world. Nature Ecol. Evol..

[45] Gaunitz C. (2018). Ancient genomes revisit the ancestry of domestic and Przewalski’s horses. Science.

[46] Randi E. (2014). Multilocus detection of wolf × dog hybridization in Italy, and guidelines for marker selection. PLoS One.

[47] Iannella A. (2019). Genetic perspectives on the historical introduction of the European rabbit (*Oryctolagus cuniculus*) to Australia. Biol. Invasions.

[48] Campbell L.G. (2009). Rapid evolution in crop-weed hybrids under artificial selection for divergent life histories. Evol. Appl..

[49] Ellstrand N.C. (2013). Introgression of crop alleles into wild or weedy populations. Annu. Rev. Ecol. Evol. Syst..

[50] Johnsson M. (2016). Feralisation targets different genomic loci to domestication in the chicken. Nat. Commun..

[51] Pan Z. (2018). Whole-genome sequences of 89 Chinese sheep suggest role of RXFP2 in the development of unique horn phenotype as response to semi-feralization. GigaScience.

[52] McGinnity P. (2003). Fitness reduction and potential extinction of wild populations of Atlantic salmon, *Salmo salar*, as a result of interactions with escaped farm salmon. Proc. R. Soc. Lond. B Biol. Sci..

[53] Hutchings J.A., Fraser D.J. (2008). The nature of fisheries-and farming-induced evolution. Mol. Ecol..

[54] Keller M. (2000). Genetic introgression from distant provenances reduces fitness in local weed populations. J. Appl. Ecol..

[55] Allendorf F.W. (2001). The problems with hybrids: setting conservation guidelines. Trends Ecol. Evol..

[56] Lynch M., O’Hely M. (2001). Captive breeding and the genetic fitness of natural populations. Conserv. Genet..

[57] Fabbri E. (2007). From the Apennines to the Alps: colonization genetics of the naturally expanding Italian wolf (*Canis lupus*) population. Mol. Ecol..

[58] Monzón J. (2014). Assessment of coyote–wolf–dog admixture using ancestry-informative diagnostic SNPs. Mol. Ecol..

[59] Pendleton A.L. (2018). Comparison of village dog and wolf genomes highlights the role of the neural crest in dog domestication. BMC Biol..

[60] Goedbloed D.J. (2013). Reintroductions and genetic introgression from domestic pigs have shaped the genetic population structure of Northwest European wild boar. BMC Genet..

[61] Linderholm A. (2016). A novel MC1R allele for black coat colour reveals the Polynesian ancestry and hybridization patterns of Hawaiian feral pigs. Royal Soc. Open Sci..

[62] Adams J. (2003). Widespread occurrence of a domestic dog mitochondrial DNA haplotype in southeastern US coyotes. Mol. Ecol..

[63] Baratti M. (2005). Introgression of chukar genes into a reintroduced red-legged partridge (*Alectoris rufa*) population in central Italy. Anim. Genet..

[64] Hegde S.G. (2006). The evolution of California’s wild radish has resulted in the extinction of its progenitors. Evolution.

[65] Heredia S.M., Ellstrand N.C. (2014). Novel seed protection in the recently evolved invasive, California wild radish, a hybrid *Raphanus* sp.(Brassicaceae). Am. J. Bot..

[66] Ridley C.E., Ellstrand N.C. (2009). Evolution of enhanced reproduction in the hybrid–derived invasive, California wild radish (*Raphanus sativus*). Biol. Invasions.

[67] Gethöffer F. (2007). Reproductive parameters of wild boar (*Sus scrofa*) in three different parts of Germany. Eur. J. Wildl. Res..

[68] Hauser T.P., Shim S.I. (2007). Survival and flowering of hybrids between cultivated and wild carrots (*Daucus carota*) in Danish grasslands. Environ. Biosaf. Res..

[69] Vandersteen W. (2012). Introgression of domesticated alleles into a wild trout genotype and the impact on seasonal survival in natural lakes. Evol. Appl..

[70] Lande R. (2015). Evolution of phenotypic plasticity in colonizing species. Mol. Ecol..

[71] Hails R.S., Morley K. (2005). Genes invading new populations: a risk assessment perspective. Trends Ecol. Evol..

[72] Wright D. (2015). Article commentary: the genetic architecture of domestication in animals. Bioinform. Biol. Insights.

[73] Wilkins A.S. (2014). The ‘domestication syndrome’ in mammals: a unified explanation based on neural crest cell behavior and genetics. Genetics.

[74] Sánchez Villagra M.R. (2016). The taming of the neural crest: a developmental perspective on the origins of morphological covariation in domesticated mammals. Royal Soc. Open Sci..

[75] Theofanopoulou C. (2017). Self-domestication in *Homo sapiens*: insights from comparative genomics. PLoS One.

[76] Pickersgill B. (2018). Parallel vs. convergent evolution in domestication and diversification of crops in the Americas. Front. Ecol. Evol..

[77] Lenser T., Theißen G. (2013). Molecular mechanisms involved in convergent crop domestication. Trends Plant Sci..

[78] Stetter M.G. (2017). How to make a domesticate. Curr. Biol..

[79] Piperno D.R. (2017). Assessing elements of an extended evolutionary synthesis for plant domestication and agricultural origin research. Proc. Natl. Acad. Sci. U. S. A..

[80] Ferrario C. (2017). Untangling the cecal microbiota of feral chickens by culturomic and metagenomic analyses. Environ. Microbiol..

[81] Robinson M.R. (2006). Live fast, die young: trade-offs between fitness components and sexually antagonistic selection on weaponry in Soay sheep. Evolution.

[82] Gratten J. (2008). A localized negative genetic correlation constrains microevolution of coat color in wild sheep. Science.

[83] Zeder M.A., Gepts P. (2012). Pathways to animal domestication. Biodiversity in Agriculture: Domestication, Evolution, and Sustainability.

[84] Jensen P. (2006). Domestication—from behaviour to genes and back again. Appl. Anim. Behav. Sci..

[85] Jensen P., Wright D., Grandin T., Deesing M.J. (2014). Behavioral genetics and animal domestication. Genetics and Behavior of Domestic Animals.

[86] Zeder M.A. (2012). The domestication of animals. J. Anthropol. Res..

[87] Kruska D.C. (2005). On the evolutionary significance of encephalization in some eutherian mammals: effects of adaptive radiation, domestication, and feralization. Brain Behav. Evol..

[88] Smith B.P. (2018). Brain size/body weight in the dingo (*Canis dingo*): comparisons with domestic and wild canids. Aust. J. Zool..

[89] Kruska D., Röhrs M. (1974). Comparative-quantitative investigations on brains of feral pigs from the Galapagos Islands and of European domestic pigs. Z. Anat. Entwicklungsgesch.

[90] Penso-Dolfin L. (2018). The evolutionary dynamics of microRNAs in domestic mammals. Sci. Rep..

[91] Ekesbo I., Gunnarsson S. (2018). Farm Animal Behaviour: Characteristics for Assessment of Health and Welfare.

[92] Rose K.M. (1985). Agonistic behaviour, responses to a novel object and some aspects of maintenance behaviour in feral-strain and domestic chickens. Appl. Anim. Behav. Sci..

[93] Swaney W.T. (2015). Behavioural responses of feral and domestic guppies (*Poecilia reticulata*) to predators and their cues. Behav. Process..

[94] Owens J.L. (2017). Visual classification of feral cat *Felis silvestris catus* vocalizations. Curr. Zool..

[95] Li C. (2006). Rice domestication by reducing shattering. Science.

[96] Thurber C.S. (2010). Molecular evolution of shattering loci in US weedy rice. Mol. Ecol..

[97] Qi X. (2015). More than one way to evolve a weed: parallel evolution of US weedy rice through independent genetic mechanisms. Mol. Ecol..

[98] Vigueira C.C. (2019). Call of the wild rice: *Oryza rufipogon* shapes weedy rice evolution in Southeast Asia. Evol. Appl..

[99] Sun J. (2019). Population genomic analysis and *de novo* assembly reveal the origin of weedy rice as an evolutionary game. Mol. Plant.

[100] MacHugh (2017). Taming the past: ancient DNA and the study of animal domestication. Annu. Rev. Anim. Biosci..

[101] Hansen S.W. (1996). Selection for behavioural traits in farm mink. Appl. Anim. Behav. Sci..

[102] Domyan E.T., Shapiro M.D. (2017). Pigeonetics takes flight: evolution, development, and genetics of intraspecific variation. Dev. Biol..

[103] Thornton E.K. (2012). Earliest Mexican Turkeys (*Meleagris gallopavo*) in the Maya region: implications for pre-Hispanic animal trade and the timing of Turkey domestication. PLoS One.

[104] Lecocq T., Teletchea F. (2018). Insects: the disregarded domestication histories. Animal Domestication.

[105] Cordain L. (1999). Cereal grains: humanity’s double-edged sword. World Rev. Nutr. Diet..

[106] Charbonneau A. (2018). Weed evolution: genetic differentiation among wild, weedy, and crop radish. Evol. Appl..

[107] Thakur M. (2018). Understanding the cryptic introgression and mixed ancestry of Red Junglefowl in India. PLoS One.

[108] Feulner P.G. (2013). Introgression and the fate of domesticated genes in a wild mammal population. Mol. Ecol..

[109] Anderson T.M. (2009). Molecular and evolutionary history of melanism in North American gray wolves. Science.

[110] Johnston S.E. (2013). Life history trade-offs at a single locus maintain sexually selected genetic variation. Nature.

[111] Glover K.A. (2017). Half a century of genetic interaction between farmed and wild Atlantic salmon: status of knowledge and unanswered questions. *Fish Fish*..

[112] Li L. (2017). Signatures of adaptation in the weedy rice genome. Nature Genet..

[113] Wu D. (2011). Genetic variation of HvCBF genes and their association with salinity tolerance in Tibetan annual wild barley. PLoS ONE.

[114] Xu L. (2015). Genome-wide association study reveals the genetic architecture of flowering time in rapeseed (*Brassica napus* L.). DNA Res..

[115] Baack E.J. (2008). Selection on domestication traits and quantitative trait loci in crop–wild sunflower hybrids. Mol. Ecol..

[116] Trut L. (2009). Animal evolution during domestication: the domesticated fox as a model. Bioessays.

[117] Mignon-Grasteau S. (2005). Genetics of adaptation and domestication in livestock. Livest. Prod. Sci..

[118] Karlsson A.C. (2016). A domestication related mutation in the thyroid stimulating hormone receptor gene (TSHR) modulates photoperiodic response and reproduction in chickens. Gen. Comp. Endocrinol..

